# Supplementary material for: Combined Metabolome and Transcriptome Analysis Revealed the Accumulation of Anthocyanins in Grape Berry (Vitis vinifera L.) under High-Temperature Stress
Source: Plants (Basel). 2024 Aug 27;13(17):2394. doi: 10.3390/plants13172394 (PMC11397361; doi:10.3390/plants13172394)
Supplement: Supplementary file 1 [file plants-13-02394-s001.zip › plants-3128687-supplementary/Supplementary/Supplementary Figure.pdf]

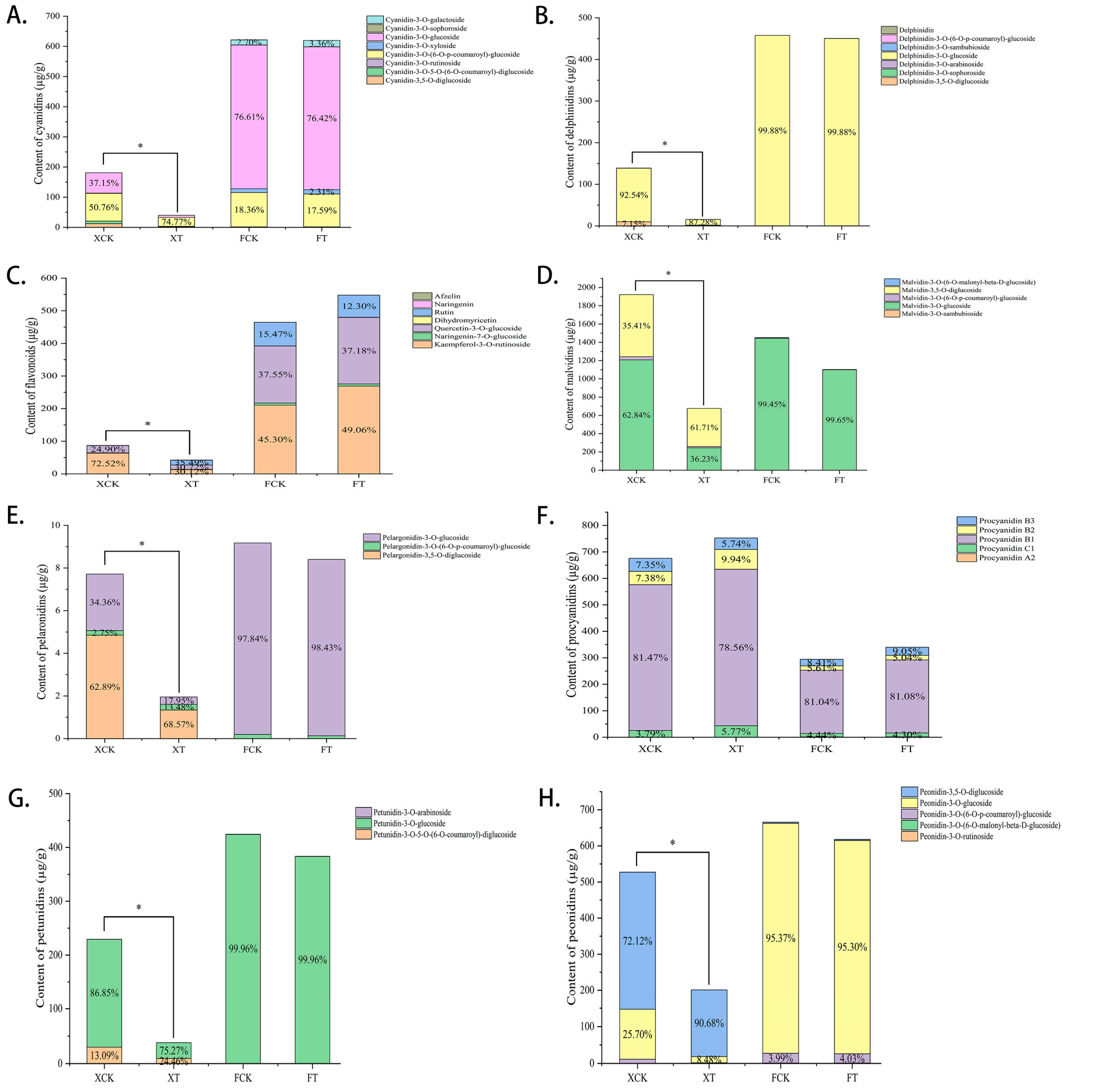

Figure S1. Column chart showing the different content of anthocyanins in HX and FL grapes at high-temperature treatment. (A) Cyanidin (B) Delphinidin (C) Flavonoid (D) Malvidin (E) Pelargonidin (F) Procyanidin (G) Petunidin (H) Peonidin.Values stand for means ± standard deviation (SDs) of three independent biological replicates. Statistical significance was measured using Student's *t*-test (* $p < 0.05$).

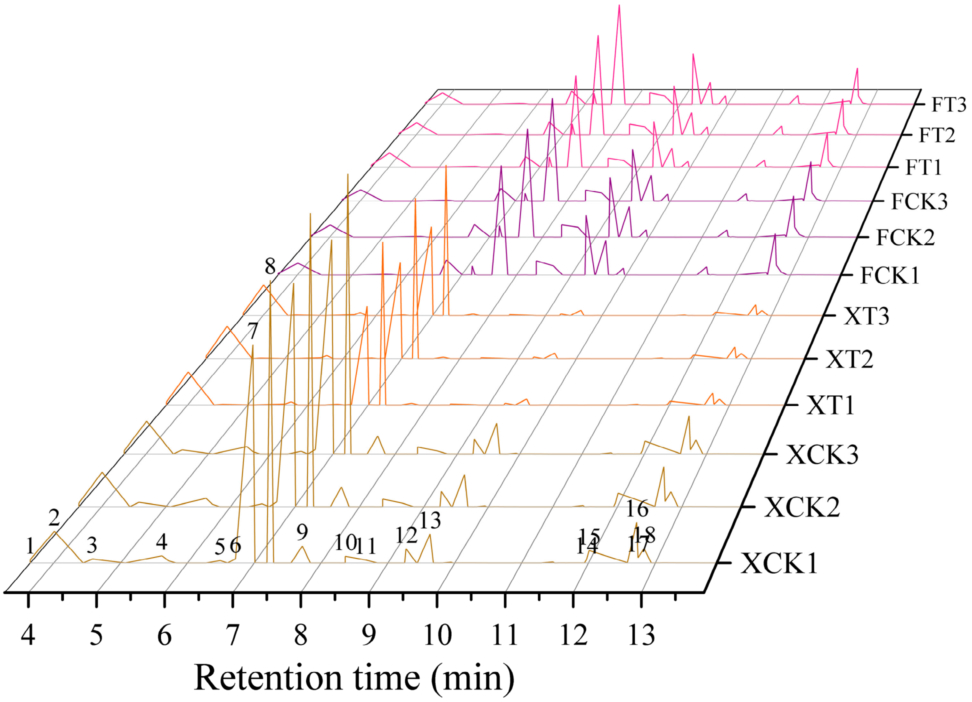

Figure S2. 'Summer Black' (XH) and 'Flame Seedless'(FL) grape berry peel anthocyanin HPLC profiles. XCK1, XCK2, XCK3, and XCK4 represent the S1, S2, S3, and S4 of XH grape in control group, respectively; XT1, XT2, XT3, and XT4 represent the S1, S2, S3, and S4 of XH grape in HT treatment group, respectively; FCK1, FCK2, FCK3, and FCK4 represent the S1, S2, S3, and S4 of FL grape in control group, respectively; FT1, FT2, FT3, and FT4 represent the S1, S2, S3, and S4 of FL grape in HT treatment group, respectively. The numbers 1-18 represent procyanidin B3, procyanidin B1, delphinidin-3,5-O-diglucoside, cyanidin-3,5-O-diglucoside, pelargonidin-3,5-O-diglucoside, delphinidin-3-O-glucoside, peonidin-3,5-O-diglucoside, malvidin-3,5-O-diglucoside, cyanidin-3-O-glucoside, petunidin-3-O-glucoside, pelargonidin-3-O-glucoside, peonidin-3-O-glucoside, malvidin-3-O-glucoside, cyanidin-3-O-5-O-(6-O-coumaroyl)-diglucoside,, petunidin-3-O-5-O-(6-O-coumaroyl)-diglucoside, cyanidin-3-O-(6-O-p-coumaroyl)-glucoside, peonidin-3-O-(6-O-p-coumaroyl)-glucoside, malvidin-3-O-(6-O-p-coumaroyl)-glucoside, respectively.

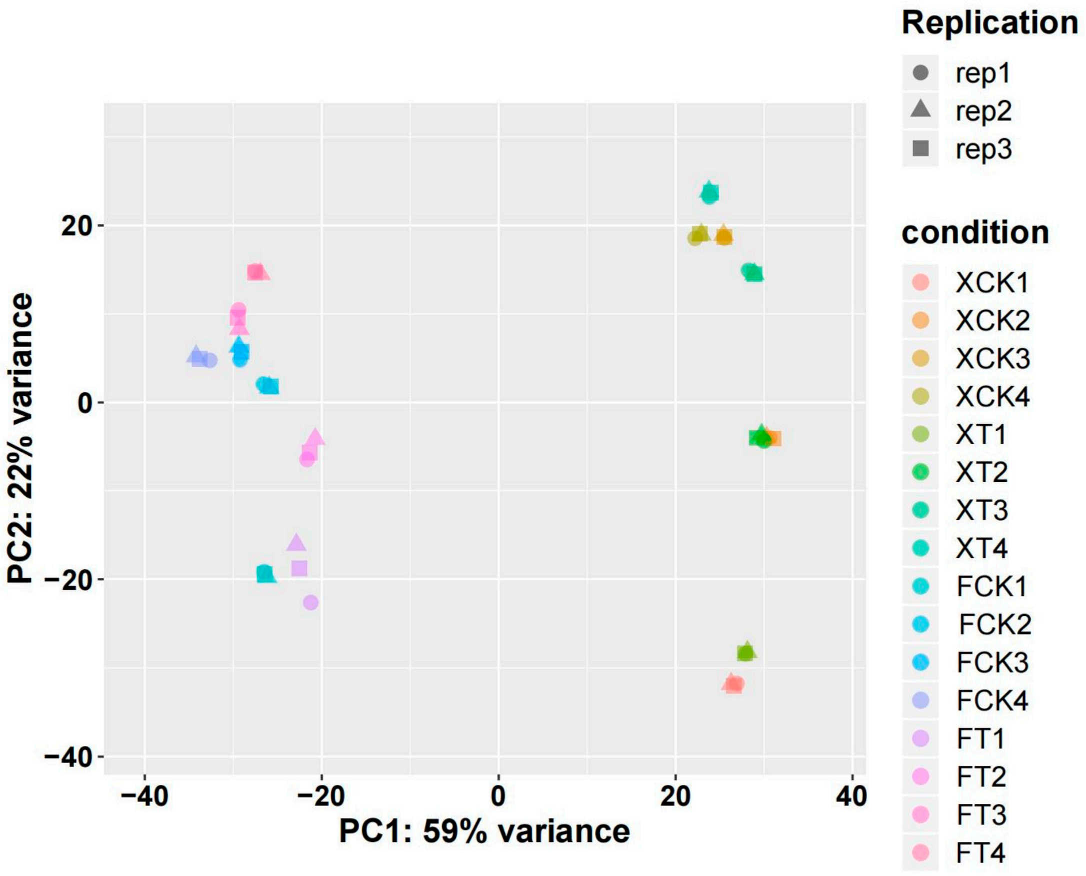

Figure S3. Scores OPLS-DA plot. XCK1, XCK2, XCK3, and XCK4 represent the S1, S2, S3, and S4 of XH grape in control group, respectively; XT1, XT2, XT3, and XT4 represent the S1, S2, S3, and S4 of XH grape in HT treatment group, respectively; FCK1, FCK2, FCK3, and FCK4 represent the S1, S2, S3, and S4 of FL grape in control group, respectively; FT1, FT2, FT3, and FT4 represent the S1, S2, S3, and S4 of FL grape in HT treatment group, respectively.

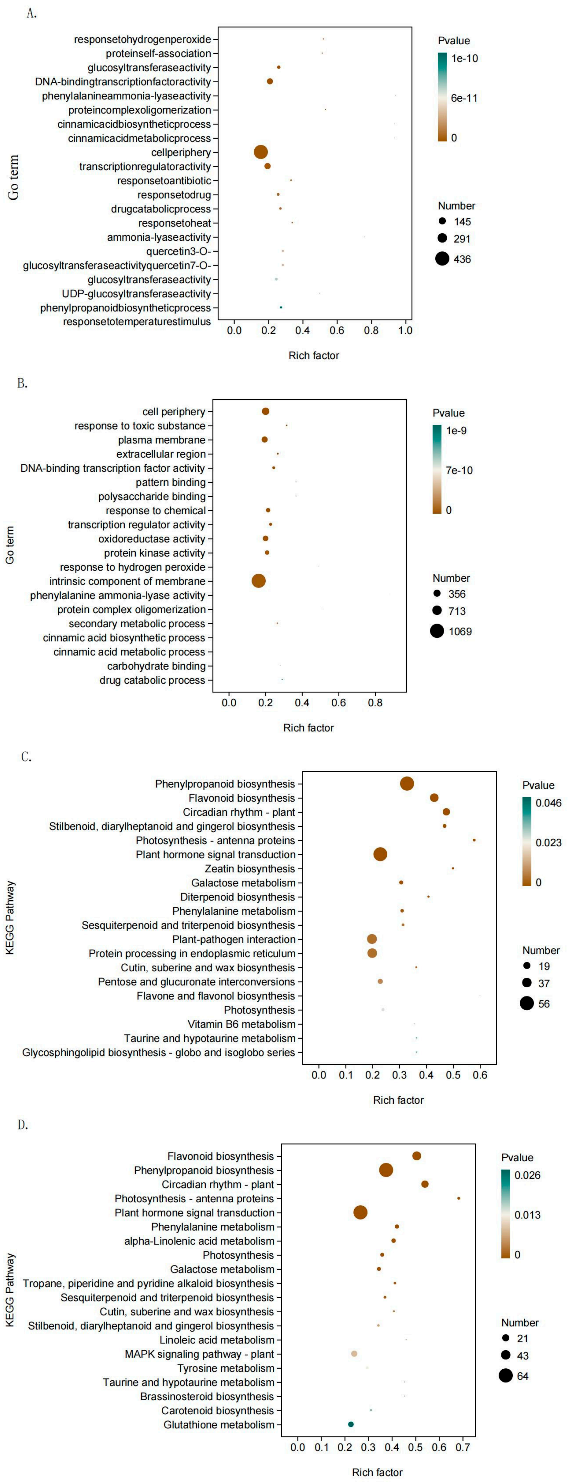

Figure S4. Statistics of GO and KEGG enrichment of DEGs under high temperature treatment in XH and FL grape peels. (A)The top 20 enriched KEGG pathways of DEGs in XCK vs XT; (B) The top 20 enriched KEGG pathways of DEGs in FCK vs FT; (B) The top 20 enriched GO terms of DEGs in XCK vs XT;(C) The top 20 enriched GO terms of DEGs in FCK vs FT; The dot size represents the number of DEGs, and dot color represents the p-value.

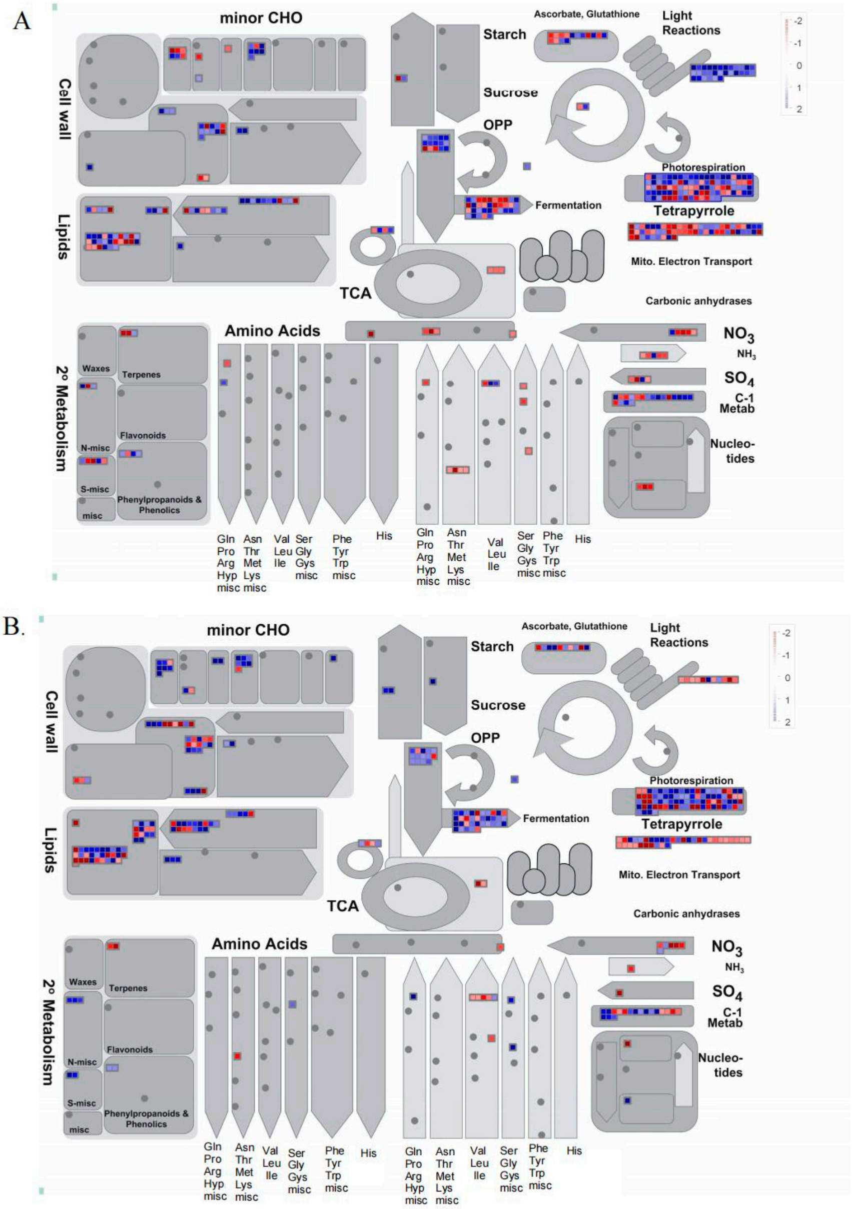

Figure S5. MapMan visualization of DEGs. Metabolic pathway with differentially expressed genes in (A) XCK vs XT, and (B) FCK vs FT.The scale bar represents the log2FC of DEGs. Red and blue indicates upregulated and downregulated genes, respectively.

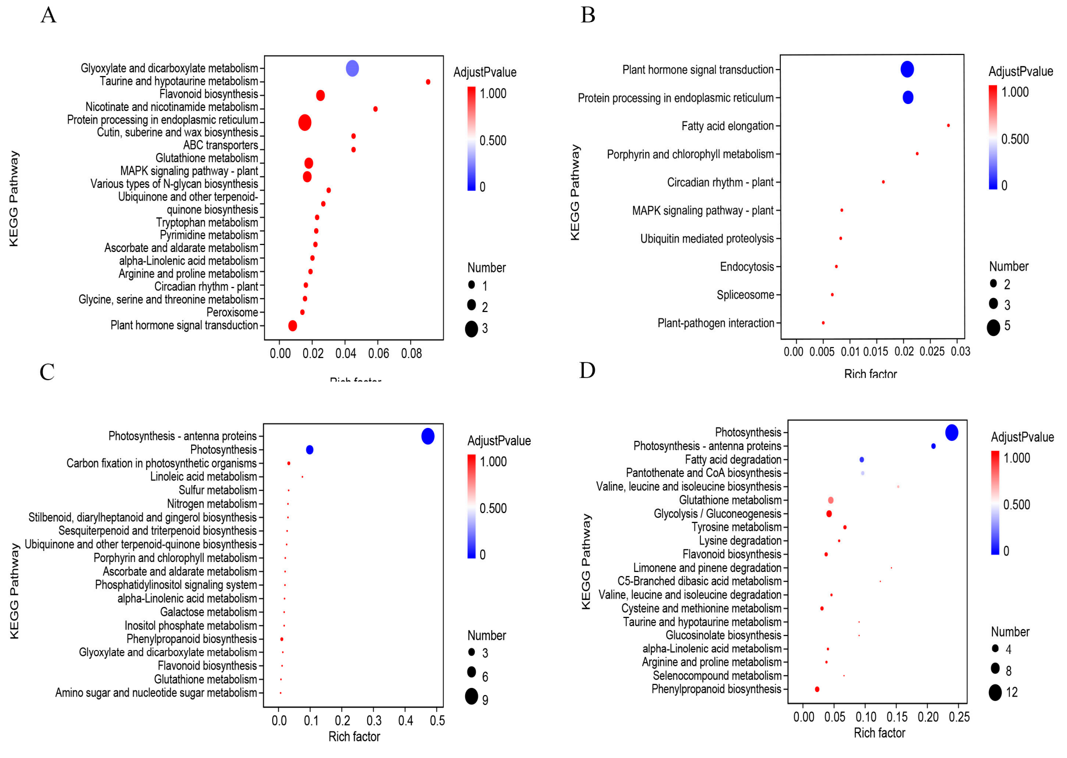

Figure S6. KEGG analysis of genes in modules at control temperature and high temperature treatment in two grape varieties.(A)The top 20 enriched KEGG pathways of DEGs in "blue" module; (B) The top 20 enriched KEGG pathways of DEGs in "megenta" module; (C) The top 20 enriched KEGG terms of DEGs in "red" module;(D) The top 20 enriched KEGG terms of DEGs in "turquoise" module; The dot size represents the number of DEGs, and dot color represents the Adjust Pvalue.

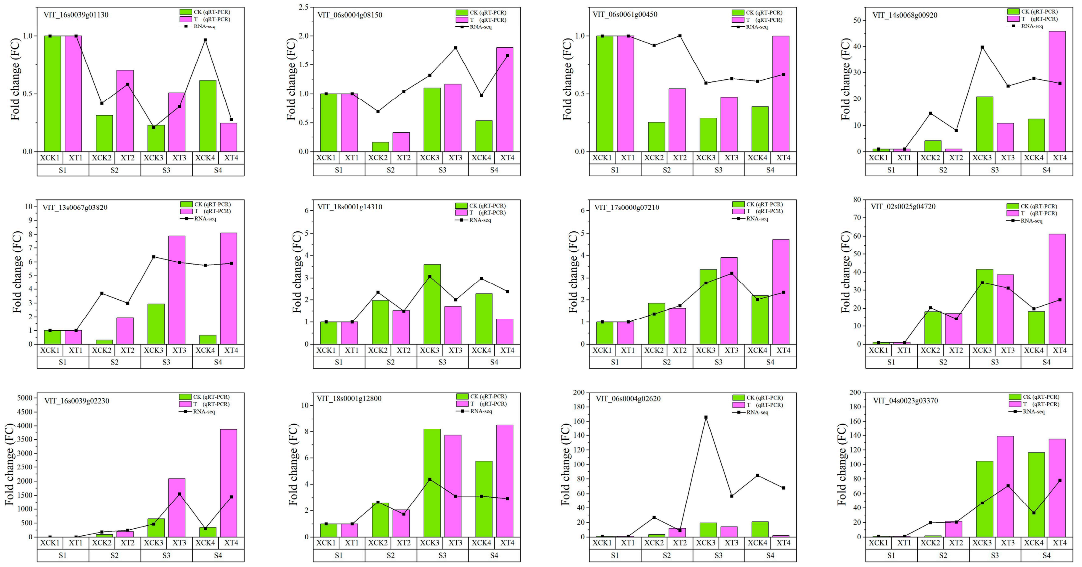

Figure S7. The fold change between qRT-PCR and RNA-seq data of XH grape. VIT_16s0039g01130: PAL. VIT_06s0004g08150: CYP73A. VIT_06s0061g00450: 4CL7. VIT_14s0068g00920: CHS. VIT_13s0067g03820: CHI. VIT_18s0001g14310: F3H. VIT_17s0000g07210: F3'H. VIT_02s0025g04720: ANS. VIT_16s0039g02230:

3GT. VIT_18s0001g12800: DFR. VIT_06s0004g02620: PAL1. VIT_04s0023g03370: F3H.

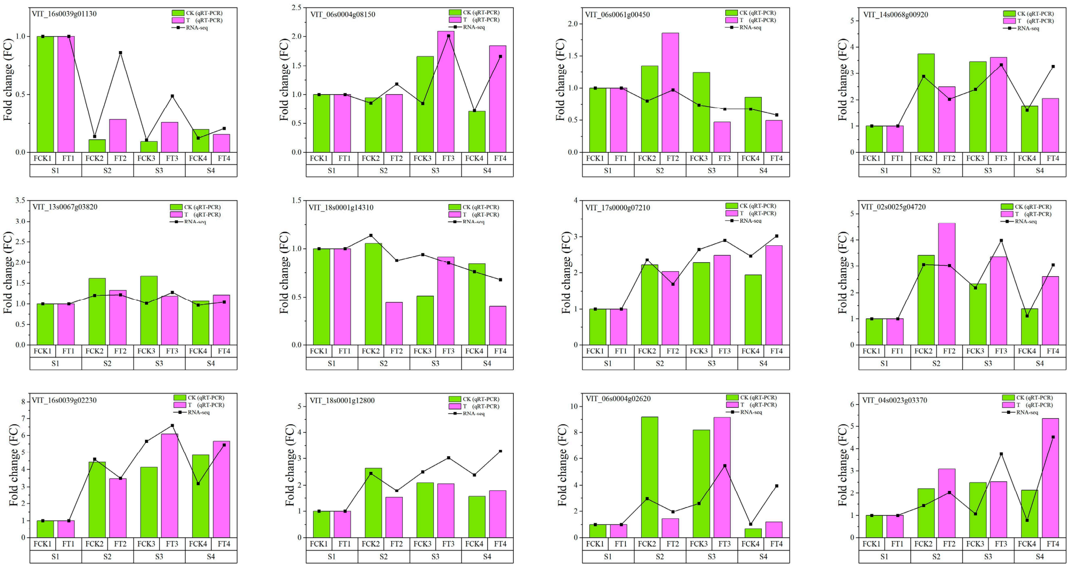

Figure S8. The fold change between qRT-PCR and RNA-seq data of FL grape. VIT_16s0039g01130: PAL. VIT_06s0004g08150: CYP73A. VIT_06s0061g00450: 4CL7. VIT_14s0068g00920: CHS. VIT_13s0067g03820: CHI. VIT_18s0001g14310: F3H. VIT_17s0000g07210: F3'H. VIT_02s0025g04720: ANS. VIT_16s0039g02230: 3GT. VIT_18s0001g12800: DFR. VIT_06s0004g02620: PAL1. VIT_04s0023g03370: F3H.
